# Improving Employee Mental Health: A Health Facility-Based Study in the United States

**DOI:** 10.3389/fpubh.2022.895048

**Published:** 2022-06-21

**Authors:** Gerald Chia Gwain, Hubert Amu, Luchuo Engelbert Bain

**Affiliations:** ^1^Department of Nursing, University of New Hampshire, Durham, DH, United States; ^2^MBI Health Services, Washington, DC, United States; ^3^Department of Population and Behavioural Sciences, School of Public Health, University of Health and Allied Sciences, Hohoe, Ghana; ^4^Lincoln International Institute for Rural Health (LIIRH), College of Social Science, University of Lincoln, Lincoln, United Kingdom

**Keywords:** depression, facility-based study, mental health, mHealth intervention, United States

## Abstract

**Background:**

In the US, over 52.9 million (21%) adults lived with a mental health illness in 2020, with depression, being one of the commonest of these conditions. The World Health Organization ranks depression as the most important contributor to global disability. As frontline workers who are responsible for taking care of a myriad of patients daily, health workers are usually exposed to depressive situations which eventually result in the development of the condition among them. This study, therefore, developed an intervention to reduce depression among workers at the Outpatient Mental Health Clinic in Washington District of Columbia, United States.

**Methods:**

A pre-intervention survey was conducted among 43 employees. The survey used the already validated Patient Health Questionnaire depression scale (PHQ-9) to determine the prevalence of depression. The WHO Healthy Workplace Model was adopted in designing an instrument for the workplace determinants of depression. An mHealth intervention was then developed and implemented among the workers. After this, a post-intervention survey was conducted among the cohort. Descriptive and inferential statistics were adopted in analyzing the data with STATA.

**Results:**

The pre-intervention survey showed a depression prevalence of 30.2% among the employees. The post-intervention survey, however, showed that the prevalence of depression among the employees reduced to 12.6%. The surveys also showed that the majority of employees who felt exposed to workplace hazards including harmful chemicals, expressed feelings of depression (pre-intervention = 53.6%; post-intervention = 80%).

**Conclusion:**

The intervention designed for this study was effective in reducing self-reported depression among employees. Improving employee mental health in health care facilities will require awareness raising among employees, mental health friendly policies, and regular follow up of employee mental health needs. Though this intervention was on a small scale, it shows promise for using cheap mhealth solutions in improving mental health at the work place.

## Introduction

Mental health disorders continue to increase globally each year (1) with over 450 million people experiencing these conditions each year (2). They are characterized by a combination of abnormal thoughts, emotions, perceptions, behavior, and relations with other conditions which include autism, bipolar disorder, dementia, schizophrenia, and depression and are responsible for 1 out of 5 years lived with disability worldwide (2). Of the 450 million people who experience mental disorders, about 264 million are due to depression alone. In the US, over 52.9 (21%) million adults lived with a mental health illness in 2020, with depression, being one of the commonest of these conditions (3).

The WHO (4) ranks depression as the most important contributor to global disability. Depression can be recurrent or long-lasting and inhibit people's ability to perform at school or the workplace as well as negatively affect overall daily life. At its peak, depression results in suicide (1, 5) with about 800,000 people committing suicide annually (6). The risk factors of depression entail multifaceted interactions between biological, social, and psychological determinants. Also, life events such as unemployment, loss, and childhood misfortune impact and may facilitate the development of depression (7).

In the United States, depression has been on the ascendency over the past two decades. The National Health Interview Survey posits that in 2019, 18.5% of adults experienced depressive symptoms that were either severe, moderate, or mild for at least 2 weeks (8). Specifically, 11.5% had mild depressive symptoms, 4.2% had moderate symptoms, and 2.8% had severe symptoms. Exacerbated by the emergence of the novel coronavirus (COVID-19) global pandemic, the prevalence of depression in the US has become over three times greater during COVID-19 than before the pandemic (9). Despite these statistics, mental health conditions including depression receive less attention compared to other health conditions in the US (10). This is because patients suffering from these conditions rarely seek help, face stigma in obtaining health care, or simply do not consider themselves to be sick (11, 12).

At the workplace, many mental health conditions, most especially depression, go undiagnosed (13). Meanwhile, prevalent undiagnosed depression can be a cause of low productivity (14). A study by Bond et al. (15) for the US Department of Health and Human Services (DHHS) indicated that depression constitutes a major leading cause of work absenteeism and responsible for high work disability insurance claims filling in both public and private sectors. The CDC reports that depression interferes with a person's ability to complete physical job tasks about 20% of the time and reduces cognitive performance about 35% of the time. Even after taking other health risks (e.g., obesity and smoking) into account, employees at elevated risk of depression had the highest healthcare costs during the 3 years after an initial health risk assessment.

Depression can be effectively managed, and people can fully recover if treatment interventions are initiated early (15). Early work-based invention is crucial because it helps mitigate devastating effects of depression and improve work performance (15). Besides, early intervention saves employees' employment and prevents consequences of unemployment including alcohol abuse, hopelessness, isolation, decreased self-esteem, increased depression and suicide and long sick leave will reduce employee's likelihood for returning to the same job (15). Active Labor Market Programmes (ALMPs), which form important components of employment support policies around the world, have also been found by the literature, to enhance mental health and wellbeing of individuals. Active Labor Market Programmes (ALMPs), which form important components of employment support policies around the world, have also been found by the literature, to enhance mental health and wellbeing of individuals (16, 17).

Short Messaging Service (SMS) and email messaging are efficient and personal forms of electronic communication, making them ideal for delivering health interventions (18). Also known as mHealth interventions, these strategies have the potential to impact mental health because cell phones and SMS/email messages are widely used around the world. mHealth interventions effectively support health behaviors and have advantages over other types of computerized interventions. Program features that improve user engagement and persuasiveness are suggested to mitigate the effect of SMS intervention (18). Our study, therefore, sought to develop an mHealth intervention to reduce the prevalence of depression among health workers. Our study is essential in that addressing depression would also mean addressing other associated health challenges that employees experience daily at work. It could, therefore, contribute effectively to the design and implementation of interventions toward addressing mental health conditions in the US.

## Materials and Methods

### Theoretical Issues

#### Introduction

The theoretical framework which guided this project was the WHO Healthy Workplace Model (19). A healthy workplace was defined as “one in which workers and managers collaborate to use a continual improvement process to protect and promote the health, safety and well-being of workers and the sustainability of the workplace by considering the following, based on identified needs: health and safety concerns in the physical work environment; health, safety and well-being concerns in the psychosocial work environment including organization of work and workplace culture; personal health resources in the workplace; and ways of participating in the community to improve the health of workers, their families and other members of the community” (19).

#### Tenets of the Theory

**Figure 1:**
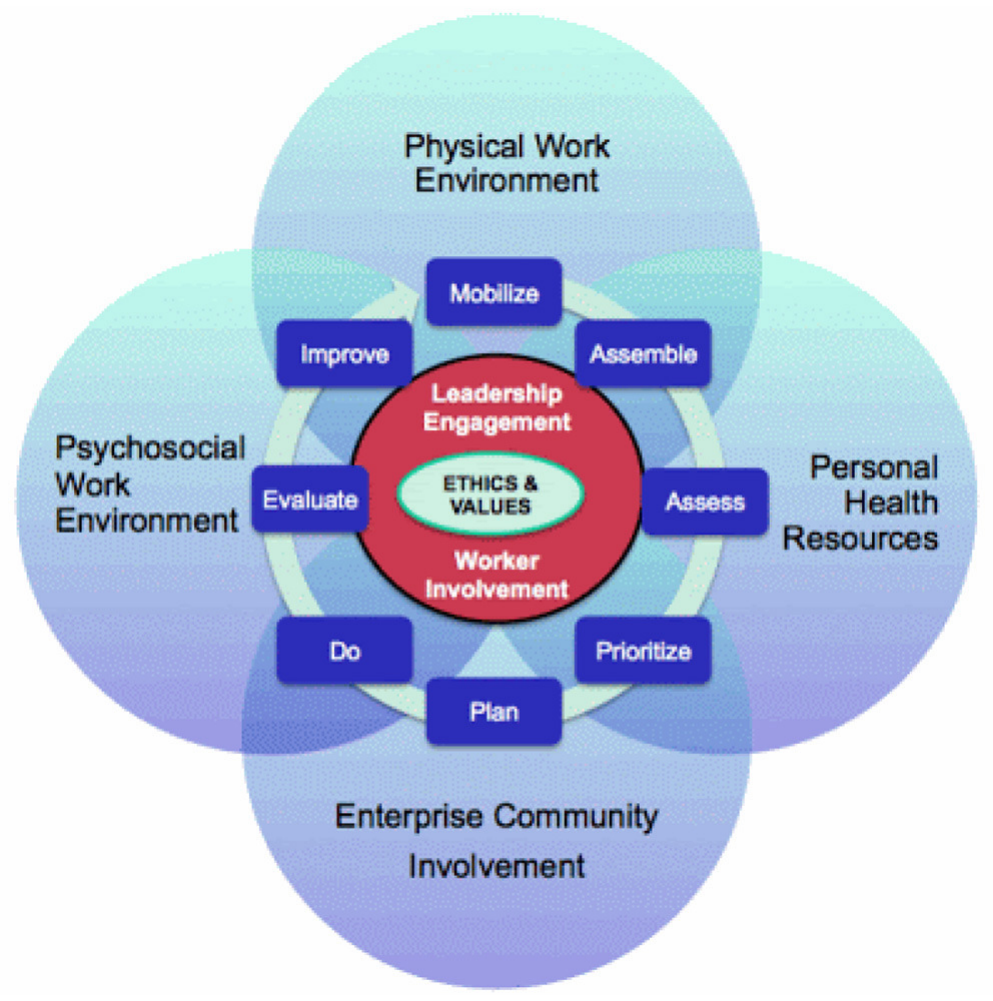
WHO Healthy Workplace Model. Source: Burton and WHO (19).

The key tenets are grouped into four large “avenues of influence.” These are the physical work environment, the psychosocial work environment, the personal health resources in the workplace, and enterprise community involvement. These avenues are not mutually exclusive entities. Rather, they overlap and influence one another (Figure 1).

The physical work environment content of the model, for instance relates to the part of the workplace facility which includes air, structure, chemicals, furniture, machines, and processes and materials present or that can happen (sometimes introducing hazards) at the workplace, and which can affect mental health including depression. The organization culture and psychosocial work environment includes practices, beliefs, values, and attitudes exhibited at work daily and which have the propensity to affect the mental health of employees leading to depression (19). Similarly, the personal health resources in the workplace (including a support environment, health services, information, and opportunities) and enterprise community involvement (including activities, expertise, and other resources a health entity engages in or provides to the environment within which it operates) could have implications for the development of depression by the employees depending on their levels of engagement in these activities/exposures. The model then offers the opportunity to integrate an intervention needed to address the identified challenges as a step toward reducing the depression levels among employees.

Rugulies (20) described a broadened perspective on the psychosocial work environment to include aspects of the job and work environment such as organizational climate or culture, work roles, interpersonal relationships at work, and the design and content of tasks (variety, meaning, scope, repetitiveness, etc.). The concept of psychosocial factors extends also to the extra-organizational environment, such as domestic demands and aspects of the individual such as personality and attitudes, which may influence the development of stress at work. Frequently, the expressions work organization or organizational factors are used interchangeably with psychosocial factors in reference to working conditions which may lead to stress (20).

#### Conclusion

The WHO Healthy Workplace Model was adopted as the conceptual framework for this study due to its relevance in outlining the essential workplace determinants of employee mental health. The model is thus, best positioned to provide the basis for the development of the factors influencing depression among health professionals in the current study. By adopting the healthy workplace model, the current study was guided in efficiently identifying the various work-related determinants of mental health and consequently the development of depression by employees.

#### Study Setting

Our study was conducted at the Outpatient Mental Health Clinic in Washington District of Columbia, US. The Outpatient Mental Health Clinic in Washington District provides services to approximately 4,000 patients. The services offered include psychiatric rehabilitative services, substance abuse and assertive community therapy programs. This project sought to create awareness of employee mental health problems and develop an intervention effective in improving the mental health of all employees at the facility and beyond.

### Study Design

#### Pre-intervention (Baseline) Survey

A baseline survey using the already validated Patient Health Questionnaire depression scale (PHQ-9) developed by Kroenke et al. (21) was carried out to examine the prevalence of depression in the workplace. To ascertain the workplace determinants of depression at the workplace, the WHO Healthy Workplace Model developed by Burton and WHO (19) was adopted. Together with the PHQ-9 and background characteristics, a questionnaire (see Appendix 1) was created and administered in the form of Google Forms among the study participants.

#### Development and Implementation of Intervention

After the baseline survey was conducted, an intervention was developed based on the findings. Specifically, already validated depression-related messages were adapted from Hartnett et al. (22) and Agyapong et al. (23) to constitute the intervention. The intervention had 8 statements as presented in Table 1.

**Table 1:** Intervention statements for employees.

**No**	**Statements**
1.	What lies behind you and what lies before you are tiny matters compared to what lies within you. Have faith in yourself and success can be yours at the work place.
2.	Letting go of resentment at the workplace is a gift you give yourself, and it will ease your professional journey immeasurably. Make peace with everyone at the work place and happiness will be yours.
3.	Pay attention to activities that have a positive impact on your mood especially at work. Note these activities and refer to them when you hit a low point to improve your mood at work.
4.	For today, focus on only what is happening now. Do not entertain negative words, thoughts or actions including those you experience at the workplace.
5.	By taking care of our physical health, our past hurts, and our present-day stresses, we can overcome low mood especially at the workplace.
6.	There are 2 days in the week we should not worry about, yesterday and tomorrow. That leaves today, live for today.
7.	Stumbling blocks can become steppingstones to a better life. You can turn adversities into opportunities.
8.	Your thoughts affect how you feel. Thoughts are not facts. Notice them and watch them come and go.

Specifically, statements 1, 2, 3, 6, and 7 were adapted from Agyapong et al. (23), while statement 4, 5, and 8 were adapted from Hartnett et al. (22). The intervention was implemented over a 1-month period (October 2021). Using text and email messaging systems, the messages were sent to the employees twice weekly. Thus, one message was sent on Tuesday and the other one was sent on Saturday. The adaptation of these messages was informed by their relative effectiveness upon implementation. The study by Hartnett et al. was a protocol for the one by Agyapong et al. In their study, Agyapong et al. carried out a single-rater-blinded randomized trial involving 73 patients with major depressive disorder. Patients in the intervention group received supportive text messages for 6 months instead of 1 month for the current study. The authors concluded by stating, “Our findings suggest that supportive text messages are a potentially useful psychological intervention for depression….”

#### Post-intervention (End-Line) Survey

Immediately after the rollout of the intervention (November 2021), a post-intervention survey was carried out to ascertain any changes in the prevalence and determinants of depression among employees. The approach adopted for the baseline survey was repeated and the initial cohort included in the survey was recruited again as part of the end-line survey. Four of the initial participants could, however, not participate in the end-line survey. As such, while the baseline survey had 43 participants, the end-line had 39 participants. The study size was informed by the small number of employees of the surveyed institution (50). As such, a census was conducted and the 43 participants were those who responded to our surveys and also took part in the intervention rollout.

#### Measurements

To measure the impact of the intervention, findings from the baseline and end-line surveys were compared. Findings from the end-line survey are useful in informing amendments/improvements to the developed intervention. Depression was categorized using a PHQ-9 score of ≥10. Socio-demographic characteristics of the participants in the pre-and post-intervention surveys were also compared. The frequency distribution of major depression and other depression by standard PHQ-9 severity intervals (0–4, 5–9, 10–14, 15–19, & 20–24) as well as the commonly used a cut-off point of ≥10. The scale was measured as Depression Severity: 0–4 none, 5–9 mild, 10–14 moderate, 15–19 moderately severe, 20–27 severe. A dichotomous variable of 0 = depressed, and 1 = Not depressed was then developed as the outcome variable. The depression instrument, background characteristics, and workplace determinants were all put into a questionnaire which was then administered during the pre- and post-intervention surveys (Appendix 1).

#### Data Analysis

Quantitative data collected from the participants were entered and cleaned using Epi data software. The data were then transported into STATA software for analysis. Data collected from the PHQ-9, socio-demographic characteristics and workplace determinants of depression were analyzed using frequency, percentage, bar charts, and chi-square analysis. Statistical significance in the chi-square analysis was determined at *p* < 0.05.

## Results

### Background Characteristics of Employees

Table 2 presents results of the background characteristics of employees included in the pre-intervention (baseline) and post-intervention (end-line) surveys. Majority of the employees in both surveys were in their 30s. Female workers constituted 55.8% in the baseline while males formed 53.9% in the end-line survey. Most of them (Pre-intervention [Baseline]: 88.4%; Post-Intervention [End-line]: 66.67%) had postsecondary/higher level of education. The majority (97 %) were Christians. The respondents were psychiatric Nurses, community support workers, and psychiatrists. The comparative majority had worked for 1–5 years in their respective professions as well as in the facility, for 1–5 years.

**Table 2:** Background characteristics of employees in the pre- and post-intervention surveys.

**Background Characteristics**	**Pre-intervention (*N* = 43) *n* (%)**	**Post-intervention (*N* = 39)** ***n* (%)**
**Age**		
<30	6 (13.95)	8 (20.51)
30–39	22 (51.16)	19 (48.72)
40–49	12 (27.91)	10 (25.64)
50+	3 (6.98)	2 (5.13)
**Sex**		
Male	19 (44.19)	21 (53.85)
Female	24 (55.81)	18 (46.15)
**Marital status**		
Never Married	16 (37.21)	17 (43.59)
Married	23 (53.49)	21 (53.85)
Divorced	3 (6.98)	1 (2.56)
Widowed	1 (2.33)	
**Educational level**		
No education	1 (2.33)	1 (2.56)
Postsecondary/higher education	38 (88.37)	26 (66.67)
Secondary	4 (9.30)	12 (30.77)
**Religion**		
Christianity	42 (97.67)	28 (97.44)
Islamic	1 (2.33)	1 (2.56)
**Occupation**		
Psychiatrists	6 (13.95)	9 (23.08)
Psychiatric Nurses	10 (23.26)	7 (17.95)
Community support workers	10 (23.26)	6 (15.38)
IT personnel	4 (9.30)	5 (12.82)
Other occupations	13 (30.23)	12 (30.77)
**Duration of practice (In years)**		
<1	6 (13.95)	6 (15.38)
1–5	19 (44.19)	18 (46.15)
6–10	14 (32.56)	10 (25.64)
11+	4 (9.30)	5 (12.82)
**Duration at facility (In years)**		
<1	10 (23.26)	10 (25.64)
1–5	22 (51.16)	16 (41.03)
6–10	10 (23.26)	10 (25.64)
11+	1 (2.33)	3 (7.69)
**Total**	**43 (100.00)**	**39 (100.00)**

### Prevalence of Reported Feelings of Depression Among Employees

**Figure 2:**
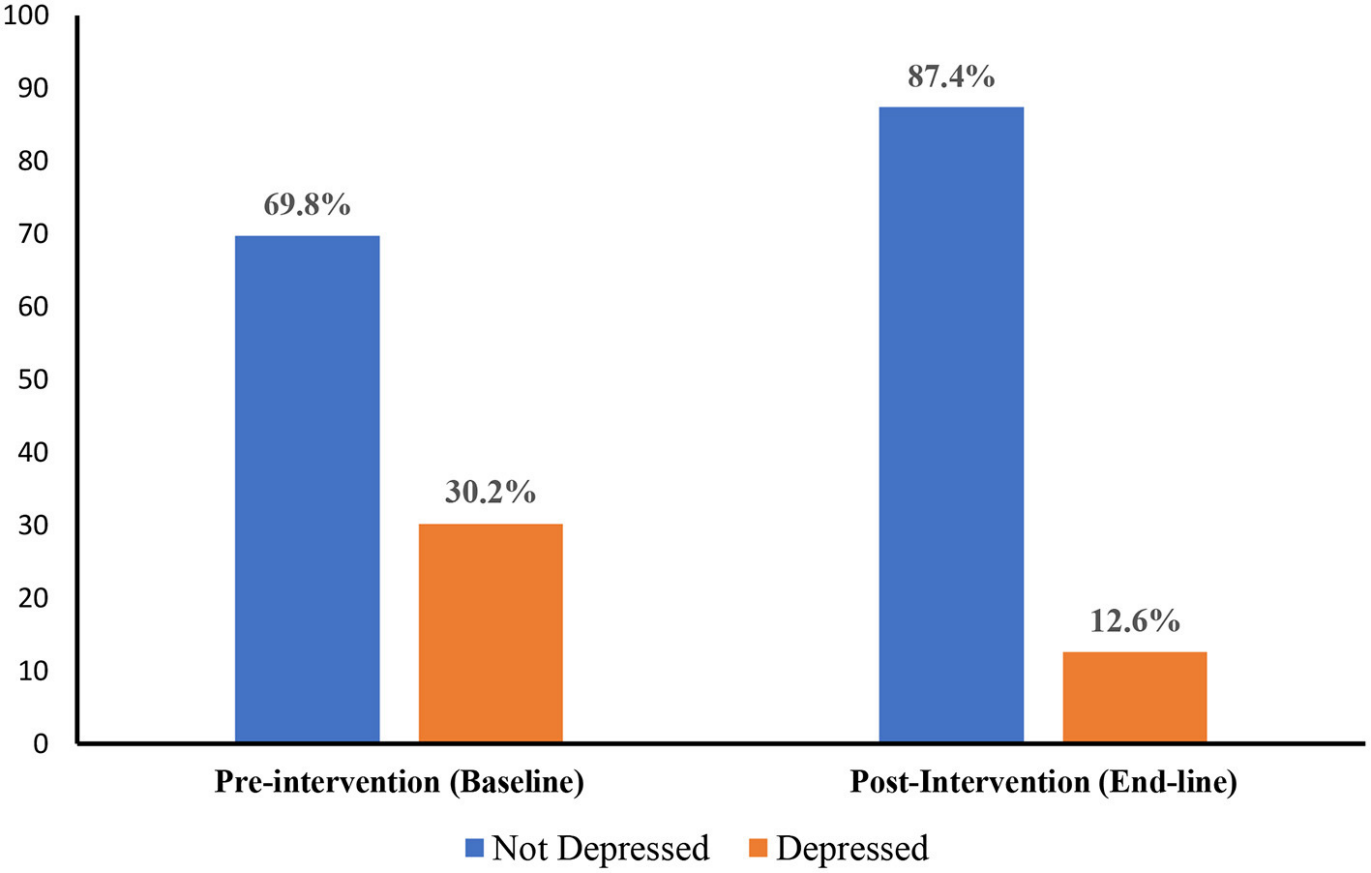
Prevalence of reported feelings of depression among employees in the pre-intervention (baseline) and post-intervention (end-line) surveys.

Depression was measured using the Personal Health Questionnaire Depression Scale (PHQ-9). Figure 2 presents results of the levels of depression in the pre-intervention and post-intervention surveys. Prior to the intervention, the prevalence of reported feelings of depression among the employees was 30.2%. This, however, declined to just 12.6% post-intervention.

### Workplace Determinants Depression Among Employees

Table 3 presents a bivariable analysis for the relationship between workplace determinants and depression among employees. In the pre-intervention survey, about 54% of workers who disagreed to the statement that they were always motivated to come to work were those who verbalized feelings of depression. Similarly, about 62% of employees who reported not being comfortable to share their mental health needs with management of their workplace also reported being feelings of depression. The majority (80%) of the employees who had healthy interpersonal relationships with their co-workers and strong and positive support environment at the workplace did not report any feelings of depression.

**Table 3:** Bivariable relationship between workplace determinants and depression.

**Workplace determinants**	**Pre-intervention survey** **(*N* = 43)**				**Post-intervention survey** **(*N* = 39)**			
	**Not depressed *n* (%)**	**Depressed** ***n* (%)**	**Chi-Square**	***p*-value**	**Not depressed *n* (%)**	**Depressed** ***n* (%)**	**Chi-Square**	***p*-value**
Always motivated to come to work			2.95	0.086			0.3123	0.576
Disagree Agree	8 (26.67) 22 (73.33)	7 (53.85) 6 (46.15)			11 (32.35) 23 (67.65)	1 (20.00) 4 (80.00)		
Air quality at the workplace is appropriate for you			0.60	0.439			0.1907	0.662
Disagree Agree	8 (26.67) 22 (73.33)	5 (38.46) 8 (61.54)			10 (29.41) 24 (70.59)	1 (20.00) 4 (80.00)		
Exposed to harmful chemicals and other environmental hazards at the workplace			0.03	0.864			1.0479	0.306
Disagree Agree	13 (43.33) 17 (56.67)	6 (46.15) 7 (53.85)			15 (44.12) 19 (55.88)	1 (20.00) 4 (80.00)		
The furniture you work with is appropriate for your health and posture			1.95	0.163			0.0025	0.960
Disagree Agree	11 (36.67) 19 (63.33)	2 (15.38) 11 (84.62)			14 (41.18) 20 (58.82)	2 (40.00) 3 (60.00)		
The attitudes of your co-workers are positive toward you			3.02	0.082			0.8434	0.358
Disagree Agree	6 (20.00) 24 (80.00)	0 (0.00) 13 (100.00)			5 (14.71) 29 (85.29)	0 (0.00) 5 (100)		
The attitude of management is positive toward you and promotes the effective discharge of your duties			0.60	0.439			0.1907	0.662
Disagree Agree	8 (26.67) 22 (73.33)	5 (38.46) 8 (61.54)			10 (29.41) 24 (70.59)	1 (20.00) 4 (80.00)		
Work-related values at the workplace are appropriate and promote your mental health			0.061	0.804			1.3358	0.248
Disagree Agree	8 (26.67) 22 (73.33)	3 (23.08) 10 (76.92)			6 (17.65) 28 (82.35)	2 (40.00) 3 (60.00)		
Strong and positive support environment at the workplace			0.60	0.443			0.0009	0.976
Disagree Agree	6 (20.00) 24 (80.00)	4 (30.77) 9 (69.23)			7 (20.59) 27 (79.41)	1 (20.00) 4 (80.00)		
Access to information is easy at your workplace			0.45	0.501			3.5511	0.060
Disagree Agree	10 (33.33) 20 (66.67)	3 (23.08) 10 (76.92)			7 (20.59) 27 (79.41)	3 (60.00) 2 (40.00)		
Access to opportunities for personal development abound at the workplace			0.22	0.642			0.0025	0.960
Disagree Agree	9 (30.00) 21 (70.00)	3 (23.08) 10 (76.92)			14 (41.18) 20 (58.82)	2 (40.00) 3 (60.00)		
Interpersonal relationships (with co-workers) at the workplace are healthy			0.13	0.721			0.6202	0.431
Disagree Agree	6 (20.00) 24 (80.00)	2 (15.38) 11 (84.62)			8 (23.53) 26 (76.47)	2 (40.00) 3 (60.00)		
Trust your co-workers when it comes to sharing your mental health needs with them?			0.34	0.559			0.4424	0.506
Disagree Agree	11 (36.67) 19 (63.33)	6 (46.15) 7 (53.85)			19 (55.88) 15 (44.12)	2 (20.00) 3 (60.00)		
The design and content of tasks are friendly at your workplace			0.28	0.596			0.1907	0.662
Disagree Agree	4 (13.33) 26 (86.67)	1 (7.69) 12 (92.31)			10 (29.41) 24 (70.59)	1 (20.00) 4 (80.00)		
Feeling that the organization takes into consideration your mental health			1.04	0.307			0.0057	0.940
Disagree Agree	9 (30.00) 21 (70.00)	6 (46.15) 7 (53.85)			13 (38.24) 21 (38.24)	2 (40.00) 3 (60.00)		
Always comfortable to share your mental health needs with management of your workplace			0.46	0.486			0.6281	0.428
Disagree Agree	15 (50.00) 15 (50.00)	8 (61.54) 5 (38.46)			20 (58.82) 14 (41.18)	2 (40.00) 3 (60.00)		
Availability and access to personal health resources at the workplace			0.003	0.960			0.0057	0.940
Disagree Agree	9 (30.0 = 0) 21 (70.00)	4 (30.77) 9 (69.23)			13 (38.24) 21 (61.76)	2 (40.00) 3 (60.00)		

In the post-intervention survey, there was no significant association between work-related determinants and depression. However, it was noted about 60.0% of workers who disagreed with the statement that access to information is easy at your workplace reported feelings of depression. The majority (85.3%) of the employees who had healthy interpersonal relationships with their co-workers at the workplace did not report any feelings of depression. Similarly, most employees (82.4%) who agreed that work-related values at the workplace were appropriate and promote their mental health did not report feelings of depression.

## Discussion

Depression is a serious mental health challenge in the US. It is a result of a complex interaction of biological, psycho-social, and psychological factors. People who have gone through unfavorable life events including unpleasant working environments have high probabilities of developing depression (24). Depression, in turn, leads to stress and dysfunction and aggravates the affected person's life. As frontline workers who are responsible for taking care of a myriad of patients daily, health workers are usually exposed to depressive situations which eventually results in them developing the mental health condition. Once that happens, interventions are required to reduce the prevalence and toll of the depressive symptoms among them. The purpose of the current project was to develop an intervention which helps to improve employee mental health in healthcare settings with focus on depression and examine workplace factors which influence depression among employees. Using the already validated Patient Health Questionnaire depression scale (PHQ-9) developed by Kroenke et al. (21) and adopting the WHO Healthy Workplace Model developed by Burton and WHO (19), a pre-intervention survey was conducted among employees of the Outpatient Mental Health Clinic in Washington District of Columbia.

Our study showed a reported feelings of depression prevalence of 30.2% among the employees. This prevalence was far more than the average depression levels in the USA as reported by the WHO (4). The survey also showed that while the work environment was generally supportive toward achieving desired mental health state, the employees felt they were exposed to workplace hazards. To address the high level of reported feelings of depression observed in the baseline survey, we designed an mHealth intervention was developed in the form of already validated depression-related messages adapted from Hartnett et al. (22) and Agyapong et al. (23). Using text messaging and email platforms, the messages were sent to employees over a one-month period within regular intervals.

Immediately after the intervention, a post-intervention (end-line) survey was carried out to assess its impact in reducing the levels of depression among the employees. The employees included in the baseline survey were the participants in the end-line survey as well. The post-intervention survey showed that the prevalence of depression among the employees had reduced to 12.6%. Chi-square analyses conducted, however, showed no statistically significant relationship between depression and the workplace determinants. Further tests of the intervention over longer durations and pre- and post-intervention surveys among higher numbers could, however, improve these associations.

The intervention demonstrated perceived improvements in mental health status through a decline in reported feelings of depression among the employees surveyed. The overall percentage change in the prevalence of between the pre- and post-intervention surveys was 17.6% which is quite significant considering the short duration within which it was carried out. Employee perception of the supportiveness of the work environment also increased, though marginally, in the post-intervention survey. This is a further indication of the effectiveness of the intervention. The study by Hartnett et al. (22) and Agyapong et al. (23) was carried out for a longer period compared to this study. They deployed supportive messages for 6 months and suggested that the text messages were a potential psychological intervention for depression in underserved population. The findings from this project following the deployment of messages for a period of 1 month showed a decrease in reported feelings of depression from 30.2% to 12.6%. This means that if the project was to expand for a period of 6 months, the results could have been more positive.

There were some limitations of the study which are worth noting. First of all, the small sample sizes used affected the significance of statistical analyses conducted. All the workplace determinants, therefore, had no statistically significant relationship with the outcome variable (depression). The intervention was carried out within one month. This to the duration of just one month between the pre- and post-intervention surveys. Given that the duration of the intervention were about three months for instance, the level of depression experienced in the post-survey could have gone further down as sustainable change usually takes time to happen. Four participants in the pre-intervention survey were lost during the follow-up survey. This could have affected the quality of comparison done in the results. While we acknowledge that the WHO healthy workplace model is appropriate in this study, there are more comprehensive literature on job quaity and mental health and which contains more workplace characteristics that are important for mental health. Our results should, therefore, be interpreted with these limitations in mind.

## Conclusion

The intervention designed for this project was effective in reducing reported feelings of depression of among employees. Following the pre-intervention and post-intervention survey, we realized that the prevalence of depression among employees declined from 30.2% to just 12.6%. Given that there is a paucity of empirical literature on workplace depression among employees in hospital settings in the US, the project has been instrumental in contributing immensely to the available literature on employee mental health.

For all health professionals in other facilities across the US, the intervention if implemented in such settings, will hopefully improve the levels of workplace related feelings of depression among them, and elevating their perceived supportiveness of the work environment. The overarching implication of this is a major contribution toward efforts at achieving the SDG 3.4 target of promoting mental health and wellbeing of all by the year 2030. The organization where the project was carried out has been experiencing a decrease in productivity. The initiation of this project was timely and caught the attention of the organization's administrators. The findings from this project were presented to the administrators including the Chief Executive Officer (CEO) of the organization.

## Data Availability Statement

The original contributions presented in the study are included in the article/Supplementary Material, further inquiries can be directed to the corresponding author.

## Ethics Statement

Ethical review and approval was not required for the study on human participants in accordance with the local legislation and institutional requirements. The patients/participants provided their written informed consent to participate in this study.

## Author Contributions

GCG conceived the study. GCG, HA, and LEB conducted the analysis and wrote the initial draft of the manuscript. All authors read the final manuscript and approved it.

## Conflict of Interest

The authors declare that the research was conducted in the absence of any commercial or financial relationships that could be construed as a potential conflict of interest.

## Publisher's Note

All claims expressed in this article are solely those of the authors and do not necessarily represent those of their affiliated organizations, or those of the publisher, the editors and the reviewers. Any product that may be evaluated in this article, or claim that may be made by its manufacturer, is not guaranteed or endorsed by the publisher.
