# Supplementary material for: Improving Employee Mental Health: A Health Facility-Based Study in the United States
Source: Front Public Health. 2022 Jun 21;10:895048. doi: 10.3389/fpubh.2022.895048 (PMC9253413; doi:10.3389/fpubh.2022.895048)
Supplement: Supplementary file 1 [file Data_Sheet_1.pdf]

Appendix A: Questionnaire

Improving Employee Mental Health: Addressing Depression at the Workplace

Section A: Background characteristics of respondents

1. How old are you?	………………
2. Sex	1 Female<br>2 Male
3. What is your religion?	1 Christianity<br>2 slam<br>3 African Traditionalist<br>4 Other [Specify]…………………
4. Marital status	1 Never married<br>2 Married<br>3 Divorced<br>4 Widowed
5. What is your highest level of education?	1 None<br>2 Elementary/primary education<br>3 Secondary education<br>4 Postsecondary / higher education
6. What is your main occupation?	1. Medical Doctor<br>2. Nurse<br>3. Midwife<br>4. Lab. Scientist<br>5. Other (Specify)…………
7. How long have you practiced in your current occupation?	………………
8. How long have you worked in the current facility?	………………

Section B: Prevalence of depression among employees (Personal Health Questionnaire Depression Scale (PHQ-9)

Over the last 2 weeks, how often have you been bothered by any of the following problems? (circle one number on each line)

**How often during the past 2 were you bothered by...**	**Not at all**	**Several days**	**More than half the days**	**Nearly every day**
1. Little interest or pleasure in doing things	0	1	2	3
2. Feeling down, depressed, or hopeless………...	0	1	2	3

3. Trouble falling or staying asleep, or sleeping too much……………………………………	0	1	2	3
4. Feeling tired or having little energy……………	0	1	2	3
5. Poor appetite or overeating…………………...…	0	1	2	3
6. Feeling bad about yourself, or that you are a failure, or have let yourself or your family down……………………………….…	0	1	2	3
7. Trouble concentrating on things, such as reading the newspaper or watching television	0	1	2	3
8. Moving or speaking so slowly that other people could have noticed. Or the opposite –being so fidgety or restless that you have been moving around a lot more than usual	0	1	2	3
9. Thoughts that you would be better off dead, or of hurting yourself in some way?	0	1	2	3

Section C: Work-related determinants of depression among employees

Circle one number on each line corresponding to your level of disagreement to the following issues at your workplace

**Workplace factors**	**Strongly Disagree**	**Disagree**	**Agree**	**Strongly agree**
1. You are always motivated to come to work	0	1	2	3
2. Air quality at the workplace is appropriate for you	0	1	2	3
3. You are not exposed to harmful chemicals and other environmental hazards at the work place	0	1	2	3
4. The furniture you work with is appropriate for your health and posture	0	1	2	3
5. The attitudes of your co-workers are positive towards you	0	1	2	3

6. The attitude of management is positive towards you and promotes the effective discharge of your duties	0	1	2	3
7. Work-related values at the workplace are appropriate and promote your mental health	0	1	2	3
8. There is a strong and positive support environment at the workplace	0	1	2	3
9. Access to information is easy at your workplace	0	1	2	3
10. Access to opportunities for personal development abound at the workplace	0	1	2	3
11. Interpersonal relationships (with co-workers) at the workplace are healthy	0	1	2	3
12. You trust your co-workers when it comes to sharing your mental health needs with them?	0	1	2	3
13. The design and content of tasks are friendly at your workplace	0	1	2	3
14. There is ready availability and access to personal health resources at the workplace	0	1	2	3
15. You have a feeling that the organization takes into consideration your mental health	0	1	2	3
16. You are always comfortable to share your mental health needs with management of your workplace	0	1	2	3

Thank you for participating in this study
